# Supplementary material for: Evaluation of the US COVID-19 Scenario Modeling Hub for informing pandemic response under uncertainty
Source: Nat Commun. 2023 Nov 20;14:7260. doi: 10.1038/s41467-023-42680-x (PMC10661184; doi:10.1038/s41467-023-42680-x)
Supplement: Supplementary file 3 — Reporting Summary [file 41467_2023_42680_MOESM3_ESM.pdf]

nature portfolio

Corresponding author(s): Emily Howerton
Last updated by author(s): Oct 6, 2023

Reporting Summary

Nature Portfolio wishes to improve the reproducibility of the work that we publish. This form provides structure for consistency and transparency in reporting. For further information on Nature Portfolio policies, see our Editorial Policies and the Editorial Policy Checklist.

Statistics

For all statistical analyses, confirm that the following items are present in the figure legend, table legend, main text, or Methods section.

n/a	Confirmed	
☐	☒	The exact sample size (*n*) for each experimental group/condition, given as a discrete number and unit of measurement
☒	☐	A statement on whether measurements were taken from distinct samples or whether the same sample was measured repeatedly
☐	☒	The statistical test(s) used AND whether they are one- or two-sided<br>*Only common tests should be described solely by name; describe more complex techniques in the Methods section.*
☐	☒	A description of all covariates tested
☐	☒	A description of any assumptions or corrections, such as tests of normality and adjustment for multiple comparisons
☐	☒	A full description of the statistical parameters including central tendency (e.g. means) or other basic estimates (e.g. regression coefficient) AND variation (e.g. standard deviation) or associated estimates of uncertainty (e.g. confidence intervals)
☒	☐	For null hypothesis testing, the test statistic (e.g. *F*, *t*, *r*) with confidence intervals, effect sizes, degrees of freedom and *P* value noted<br>*Give P values as exact values whenever suitable.*
☒	☐	For Bayesian analysis, information on the choice of priors and Markov chain Monte Carlo settings
☒	☐	For hierarchical and complex designs, identification of the appropriate level for tests and full reporting of outcomes
☒	☐	Estimates of effect sizes (e.g. Cohen's *d*, Pearson's *r*), indicating how they were calculated

Our web collection on statistics for biologists contains articles on many of the points above.

Software and code

Policy information about availability of computer code

Data collection	Individual modeling teams submitted projections to the public GitHub repository https://github.com/midas-network/covid19-scenario-modeling-hub.
Data analysis	All analyses were performed in R, version 4.2.0. All necessary data and code to reproduce analyses are available at https://github.com/midas-network/covid19-scenario-hub_evaluation/ and deposited at https://zenodo.org/record/8415147. For a complete list of packages used, and corresponding versions, see renv.lock file in this repository.

For manuscripts utilizing custom algorithms or software that are central to the research but not yet described in published literature, software must be made available to editors and reviewers. We strongly encourage code deposition in a community repository (e.g. GitHub). See the Nature Portfolio guidelines for submitting code & software for further information.

Data

Policy information about availability of data

All manuscripts must include a data availability statement. This statement should provide the following information, where applicable:

- Accession codes, unique identifiers, or web links for publicly available datasets
- A description of any restrictions on data availability
- For clinical datasets or third party data, please ensure that the statement adheres to our policy

All data analyzed in the present study can be viewed online at https://covid19scenariomodelinghub.org/ and downloaded at https://github.com/midas-network/covid19-scenario-hub_evaluation/tree/main/data-raw.

Research involving human participants, their data, or biological material

Policy information about studies with human participants or human data. See also policy information about sex, gender (identity/presentation), and sexual orientation and race, ethnicity and racism.

Reporting on sex and gender	No human participants or data was used in this study.
Reporting on race, ethnicity, or other socially relevant groupings	No human participants or data was used in this study.
Population characteristics	No human participants or data was used in this study.
Recruitment	No human participants or data was used in this study.
Ethics oversight	No human participants or data was used in this study.

Note that full information on the approval of the study protocol must also be provided in the manuscript.

Field-specific reporting

Please select the one below that is the best fit for your research. If you are not sure, read the appropriate sections before making your selection.

☐ Life sciences ☐ Behavioural & social sciences ☒ Ecological, evolutionary & environmental sciences

For a reference copy of the document with all sections, see nature.com/documents/nr-reporting-summary-flat.pdf

Ecological, evolutionary & environmental sciences study design

All studies must disclose on these points even when the disclosure is negative.

Study description	This modeling study assesses the performance of the first 16 rounds of COVID-19 Scenario Modeling Hub (SMH) projections.
Research sample	SMH is a collaborative hub that collected projections of COVID-19 cases, hospitalizations, and deaths from multiple, independent modeling teams. SMH used an open call to solicit projections that met baseline formatting criteria from any group. Teams made projections for four distinct future scenarios, and projections ranged from 3-month ahead to 12-month ahead, depending on round-specific goals.
Sampling strategy	This study assessed all available SMH projections made through November 2022. Bootstrap intervals were calculated based on 1,000 samples, which proved in initial tests to be large enough to generate stable intervals.
Data collection	Data analyzed here are individual projections from the COVID-19 Scenario Modeling Hub. Individual modeling teams submitted these projections via pull request to the public SMH GitHub repository (https://github.com/midas-network/covid19-scenario-modeling-hub).
Timing and spatial scale	This study assesses projections made between December 2020, when the COVID-19 Scenario Modeling Hub was founded, and November 2022, the most recent set of projections at the time of submission.
Data exclusions	Submitted projections that did not comply with basic SMH requirements (e.g., probabilistic estimates) were excluded from this analysis. A detailed description of all exclusions is provided in Table S2.
Reproducibility	This modeling study can be fully reproduced using data and code available at https://github.com/midas-network/covid19-scenario-hub_evaluation/.
Randomization	This retrospective analysis aimed to assess the performance of all SMH projections. SMH accepted projections from all groups willing

Randomization	to participate, and generated projections at times when those projections would answer timely public health questions. Therefore, randomization is not relevant.
Blinding	SMH teams submit projections independently to avoid biases such as group-think. Team names are anonymized in all figures for this manuscript.

Did the study involve field work? ☐ Yes ☒ No

Reporting for specific materials, systems and methods

We require information from authors about some types of materials, experimental systems and methods used in many studies. Here, indicate whether each material, system or method listed is relevant to your study. If you are not sure if a list item applies to your research, read the appropriate section before selecting a response.

Materials & experimental systems

n/a	Involved in the study
☒	☐ Antibodies
☒	☐ Eukaryotic cell lines
☒	☐ Palaeontology and archaeology
☒	☐ Animals and other organisms
☒	☐ Clinical data
☒	☐ Dual use research of concern
☒	☐ Plants

Methods

n/a	Involved in the study
☒	☐ ChIP-seq
☒	☐ Flow cytometry
☒	☐ MRI-based neuroimaging
